# Enhancing the electro-mechanical properties of polydimethylsiloxane elastomers through blending with poly(dimethylsiloxane-*co*-methylphenylsiloxane) copolymers†

**DOI:** 10.1039/c8ra02314j

**Published:** 2018-06-25

**Authors:** Peter Jeppe Madsen, Liyun Yu, Sarah Boucher, Anne Ladegaard Skov

**Affiliations:** Danish Polymer Centre, Department of Chemical and Biochemical Engineering, Technical University of Denmark Søltofts Plads Building 227 2800 Kgs. Lyngby Denmark al@kt.dtu.dk +45 45882258 +45 45252825

## Abstract

In this work, improved electro-mechanical properties of silicone-based dielectric elastomers are achieved by means of adding so-called “voltage-stabilisers” prepared from phenyl-functional copolymers prepared using oxyanionic ring-opening polymerisation of octamethylcyclotetrasiloxane (D4) and either tetramethyltetraphenylcyclotetrasiloxane (T4) or octaphenylcyclotetrasiloxane (O4). The concentration of the voltage stabiliser was varied both by changing the molar ratio between methyl and phenyl groups in the copolymer and also by varying the amount of copolymer mixed into a PDMS-based elastomer. The phenyl-functional copolymers were generally found to disperse homogeneously in the PDMS matrix and this resulted in networks with improved mechanical and electrical properties. The developed elastomers were inherently extensible with enhanced tensile and tear strengths, due to phenyl-rich microphases acting as reinforcing domains. Furthermore, addition of phenyl-functional copolymers resulted in elastomers with increased relative permittivity and electrical breakdown strength compared to control elastomers while retaining a low dielectric loss. This demonstrates their efficiency as voltage stabilisers.

## Abstract 2

Improved electro-mechanical properties of silicone-based dielectric elastomers are achieved by means of adding so-called “voltage-stabilisers” prepared from phenyl-functional copolymers prepared using oxyanionic ring-opening polymerisation.

## Graphical abstract

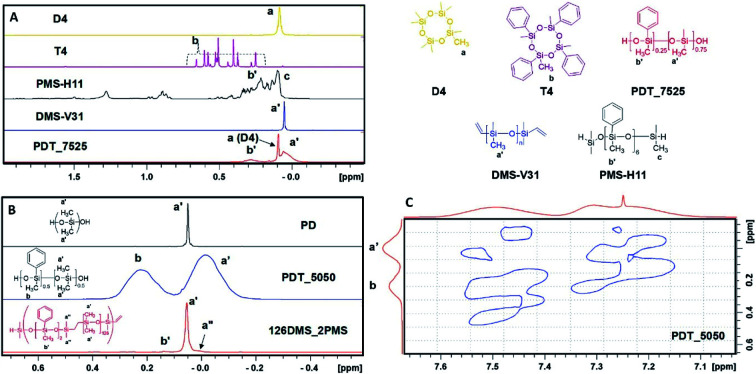

## Introduction

Dielectric elastomers (DEs) hold great promise as materials for novel, advanced electromechanical applications such as actuators, generators and sensors, due to their simple, linear and flexible working principle combined with the promise of lightweight and cheap transducers.^1–7^ Originally they were nick-named artificial muscles, and various DE-based implants have been developed over the years, such as artificial eyelid controllers^8^ and sphincters.^9,10^ However, DEs have found broader applications in general, such as in microfluidic flow control,^11^ optical lenses,^12^ loudspeakers,^13^ wave energy harvesters,^14^ window frames with haze function^15^ and haptics,^16^ to mention just a few original ideas for applications currently under investigation or under commercialization.

This broad applicability comes down to some of the main characteristics of dielectric elastomers, namely that they possess high extensibility, flexibility and vanishing mechanical fatigue as well as high electrical and mechanical breakdown strengths.^17^

As discussed in a recent review by Skov *et al.*^18^ on methodologies to improve the electro-mechanical properties of dielectric elastomers, methodologies fall under the following categories: (1) silicone composites,^19–26^ (2) silicone/polymer blends,^27–31^ (3) chemically modified silicones,^32–35^ and (4) systems with a complex network structure.^36–41^ Simply blending in functional polymers of various compositions, structures and/or molecular weights is an attractive approach because it is relatively simple to optimise and scale up.^27^ However, it requires miscibility for product reliability, and it is therefore not trivial for silicones, which are immiscible with most other polymers. One of the earliest examples of crosslinked blends used as dielectric elastomers consists of an addition-curing silicone elastomer with dissolved poly(hexylthiophene) for improved dielectric permittivity.^27^ However, the system has two flaws, namely that the two types of polymers are completely immiscible, and the sulphur in poly(hexylthiophene) furthermore inhibits the catalyst (Pt) required for the curing mechanism. In other words, it means that the network structure in this case is limited and that the blend is not thermodynamically stable and will phase separate over time. Dielectric elastomer stability is attracting a lot of attention, and various methods have been proposed recently.^28,29,42–44^ All these methods will easily detect phase separation since it will affect all properties largely.

Choosing the right polymer for the blending approach is therefore of utmost importance.

Voltage stabilisers are well known in the high-voltage cable industry, where most interest has centred on stabilising (*i.e.* increasing the electrical breakdown strength of) polyethylene (PE), the most commonly used material for high-voltage cables.^45–47^ For PE the addition of polar co-monomers was found to alter the electrical breakdown strength, which was found to go through a maximum at around 0.1% mol mol^−1^.^48^ The mechanism and optimisation of voltage stabilisers has also been treated theoretically.^49^ A study in our laboratories shows that sub-percentage additions of various aromatic substances can increase electrical breakdown strength significantly *via* voltage stabilisation, due to an electron trapping effect.^50–52^ Voltage stabilisation of silicone elastomers was achieved by replacing some methyl groups of PDMS-based elastomers with phenyl groups (resulting in multiblock elastomers based on PDMS-*co*-poly(phenylmethylsiloxane) (PPMS) elastomers).^51^ Additionally, combining two copolymers (PDMS-polyethyleneglycol (PEG) and PDMS-PPMS multiblock copolymers) enhanced both relative permittivity and electrical breakdown strength. However, the ultimate stresses of these elastomers were significantly smaller than those of common PDMS elastomers.^52^ From these results, it was obvious that phase separation had a beneficial impact on voltage stabilisation. Therefore, it is important to be able to control the compatibility between the two components in order to control the domain size.

There is still large room for improvement for silicone elastomers compared to the results achieved for PE.

In this work, the modification of silicone elastomers is explored further by adding random PDMS-*co*-PPMS and PDMS-poly(diphenylsiloxane) with varying ratios between methyl and phenyl to a PDMS matrix. Material properties such as relative permittivity and electrical breakdown strength, as well as tensile and tear strengths, are mapped as a function of phenyl concentration in the matrix. All elastomers are furthermore tested after ten months storage in order to ensure that the elastomers are thermodynamically stable. These methodical studies are believed to be highly useful for tailoring dielectric elastomers with specific electrical and mechanical properties.

## Experimental

### Materials

Octamethylcyclotetrasiloxane (D4) was acquired from TCI, Belgium, and dried over molecular sieves prior to use. Octaphenylcyclotetrasiloxane (O4) was acquired from TCI and used as received. Tetramethyltetraphenylcyclotetrasiloxane (T4, mixture of isomers) and dimethylformamide (DMF) were acquired from Sigma-Aldrich and dried over molecular sieves prior to use.

Potassium hydroxide was purchased from Sigma-Aldrich as pellets. Prior to use, these were refluxed under cyclohexane at 110 °C for at least 48 h. The resulting suspension was filtered and the precipitate was dried in a vacuum oven.

Vinyl-terminated PDMS, DMS-V31 (*M*_n_ = 28 000 g mol^−1^) and a hydride-functional cross-linker, HMS-301 (*M*_n_ = 1950 g mol^−1^), were acquired from Gelest Inc, USA. The platinum cyclovinylmethyl siloxane complex catalyst (511) was purchased from Hanse Chemie, Germany, while silicon dioxide amorphous hexamethyldisilazane-treated particles (SIS6962.0) were purchased from Fluorochem, Belgium. The inhibitor Pt88 was acquired from Wacker Chemie AG, and all other chemicals were acquired from Sigma-Aldrich, Denmark, and used as received, unless otherwise stated.

### Synthesis of polydimethylsiloxane (PD) and poly(dimethylsiloxane-*co*-phenylmethylsiloxane) (PDT)

All glassware was dried in a 130 °C oven for at least 24 h prior to use. All polymers were prepared following a procedure based on literature protocols.^53–55^ In a typical procedure, a Schlenk tube was charged with potassium hydroxide (0.051 g, 0.90 mmol), which was then fitted with a septum. This was evacuated and refilled with nitrogen three times. Then, D4 (10.4 mL, 9.8 g, 33 mmol) and DMF (0.1 mL, 0.095 g, 1.3 mmol) were introduced into the Schlenk tube, using nitrogen-purged syringes. The resulting solution was placed in a 60 °C oil bath for 1.5 h. After cooling to room temperature, 0.1 mL acetic acid was added to quench the reaction, and the highly viscous product was transferred to a vial. The polymer was then dried in a vacuum oven and used without further purification. For copolymers of D4 and T4, the dried monomers were introduced simultaneously into the Schlenk tube in the desired ratio.

### Synthesis poly(dimethylsiloxane-*co*-diphenylsiloxane) (PDO) copolymer

The general procedure described above was followed, except that O4 (3.56 g, 4.5 mmol) and potassium hydroxide (0.055 g, 1 mmol) were introduced in the Schlenk tube prior to evacuation and refilling with nitrogen. Dried D4 (4.04 g, 13.6 mmol) and DMF (0.1 mL, 0.095 g, 1.3 mmol) were then introduced. It was necessary to heat the mixture to 90 °C to obtain a homogenous polymerisation mixture. The polymerisation was terminated after 1.5 h and treated as described above.

### Preparation of elastomer films

PDT or PDO, vinyl-terminated PDMS, DMS-V31, and an 8-functional cross-linker HMS-301 were mixed with treated silica particles (25 wt%) and an inhibitor (1 wt%, Pt88) and then mixed on a FlackTek Inc. DAC 150.1 FVZ-K SpeedMixer™. For the control, neither PDT nor PDO was added. The catalyst (511) (1.5 ppm) was added thereafter and the mixture was speed-mixed once more. The uniform mixture was then poured into a 1 mm-thick steel mould and furthermore coated as 150 μm films on a glass substrate, using a film applicator (3540 bird, Elcometer, Germany), and fully cured at 80 °C for 12 hours. The added amounts of copolymer are given in units of phr (parts per hundred rubber, *i.e.* g per 100 g of elastomer basis).

### Characterisation

#### Nuclear magnetic resonance spectroscopy (NMR)

^1^H and NOESY NMR experiments were performed on a Bruker 300 MHz spectrometer as 100 mg mL^−1^ solutions in CDCl_3_. Data were analysed using TopSpin version 3.5 pl 7 from Bruker.

#### Size exclusion chromatography (SEC)

Size exclusion chromatography (SEC) was performed on a modular SEC system consisting of a Shimadzu LC-10AD VP pump, a Shimadzu SIL-10AD VP autosampler coupled to two PSS SDV Linear columns (8 × 300 mm) in series and a Shimadzu RID-10A RI detector. Samples were run in toluene at an ambient temperature (22 °C) at a flow rate of 1 mL min^−1^. Molar mass characteristics were calculated using OMNISEC software version 5.10.46.1. The system was calibrated using linear polydimethylsiloxane (PDMS) standards acquired from PSS.

#### Determination of gel fractions

The gel fractions were determined using gel extraction, whereby a film sample (∼200 mg) was immersed in chloroform (∼10 mL) at room temperature for 48 h. The chloroform was replaced after 24 h. The solvent was decanted off and the films were washed several times with fresh solvent. The samples were dried for 48 h at room temperature under ambient pressure until the weight was constant. Gel fractions were calculated as the weight after extraction and drying (*m*_e_) against the initial weight of the sample (*m*_0_) as *W*_gel_ = *m*_e_/*m*_0_. The extracts were allowed to evaporate and analysed by ^1^H NMR and size exclusion chromatography.

#### Measurement of shear moduli

Rheological characterisation of the prepared films was performed with a TA Instruments 2000 Rheometer set to 2% controlled strain mode, which was ensured to be within the linear viscoelastic regime by conducting initial strain sweeps. Measurements were performed with parallel plate geometry of 25 mm at room temperature, with a normal force of ∼7 N and in the frequency range 100–0.01 Hz.

#### Measurement of Young's modulus, tensile and tear strength

Uniaxial extensional rheology was performed on the series of elastomer films in order to determine the Young's modulus and tensile strength, as well as tear strength. The stress–strain curves of the films were recorded at room temperature with an ARES-G2 rheometer, using the SER2 geometry. A specimen 20 mm in length and 6 mm wide was placed between two drums and initially separated by a distance of 12.7 mm. The test specimen was elongated uniaxially at a steady Hencky strain rate of 0.01 s^−1^ until sample failure in the middle part. Each composition was subjected to four tensile measurements, which were then averaged. Young's moduli were obtained from the tangent of the stress–strain curves at 5% strain.

A sample for tear strength measurement was made from a cutting die according to ASTM D 624 B with a 0.5 mm-deep nick in the middle. The test specimen was elongated uniaxially at a steady Hencky strain rate of 0.01 s^−1^ until it was completely torn. Each composition was subjected to four tensile measurements, which were then averaged.

#### Scanning electron microscopy (SEM)

The morphology of the films was investigated with an FEI Quanta 200 ESEM scanning electron microscope, equipped with a field emission gun. The films were coated in 2 nm-thick gold by means of a sputter coater (Cressington, model 208HR) under vacuum conditions and a current of 40 mA for 5 seconds. The sample surface was detected with a secondary electron detector (ETD SE) for an incident electron beam of spot 3.5 accelerated to 15–20 keV.

#### Dielectric properties determination

Dielectric relaxation spectroscopy (DRS) was performed on a Novocontrol Alpha-A high-performance frequency analyser (Novocontrol Technologies GmbH & Co) operating in the frequency range 10^−1^ to 10^6^ Hz at room temperature and in a low electrical field (∼1 V mm^−1^). The diameter of the tested samples was 25 mm and their thicknesses were in the range of 0.5 to 1 mm.

#### Electrical breakdown strength determination

Electrical breakdown tests were performed on an in-house-built device based on international standards (IEC 60243-1 (1998) and IEC 60243-2 (2001)).^20^ Film thicknesses were measured through microscopy of cross-sectional cuts. The distance between the spherical electrodes was set accordingly using a micrometer stage and gauge. An indent of less than 5% of sample thickness was then applied to ensure that the spheres were in contact with the sample. The polymer film was slid between the two spherical electrodes (diameter of 20 mm), and the breakdown strength was measured at the point of contact by applying a stepwise increasing voltage (50–100 V step^−1^) at a rate of 0.5–1 steps s^−1^. Each sample was subjected to 12 breakdown measurements at room temperature, and an average of these values was given as the breakdown strength of the sample.

## Results and discussion

**Scheme 1:**
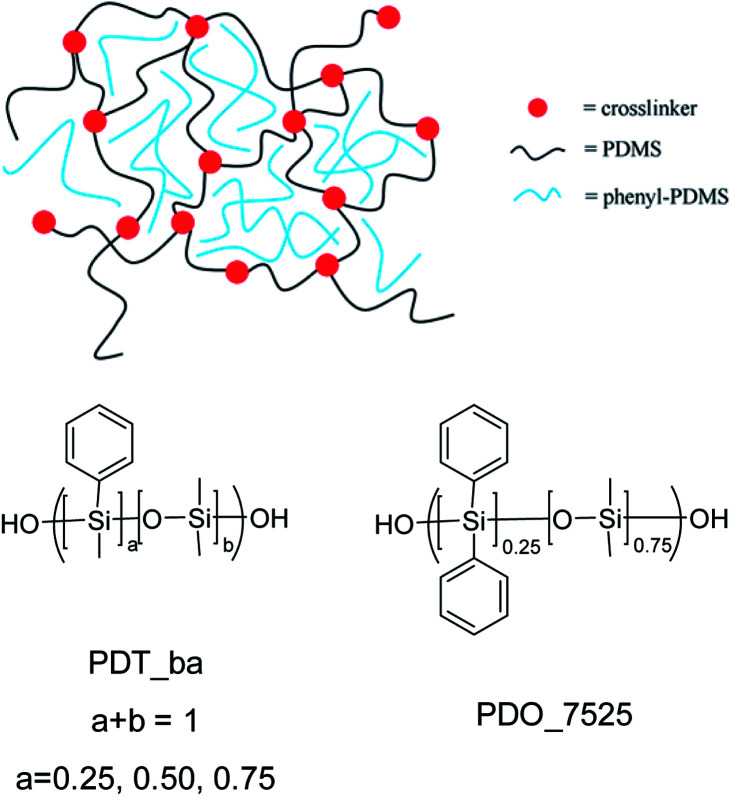
Cross-linked elastomer consisting of phenyl-PDMS copolymer in PDMS matrix and structures of the added phenyl-PDMS copolymers.

The result of mixing elastomers with phenyl-PDMS copolymers is illustrated in Scheme 1. The PDMS chains constituting the matrix have reactive groups that facilitates crosslinking. The phenyl-PDMS copolymers do not have reactive groups, but will form phenyl-rich domains with a size that is mainly governed by the compatibility between the copolymers and the matrix. The general structures of the phenyl-PDMS copolymers are also shown in Scheme 1. These vary in relative phenyl content and in amount of phenyl substituents on silicium. This approach allows for systematic variation of overall phenyl content and density.

### Polymer synthesis

**Scheme 2:**
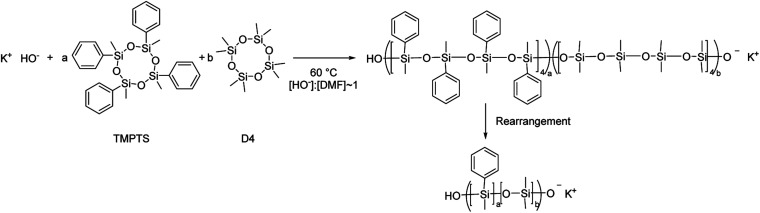
Anionic ring-opening polymerisation of cyclotetrasiloxanes in the presence of *N*,*N*-dimethylformamide as a polymerisation promoter.

The oxyanionic homopolymerisation of octamethylcyclotetrasiloxane (D4) and copolymerisation of octamethylcyclotetrasiloxane and tetramethyltetraphenylcyclotetrasiloxane (T4) was carried out at 60 °C in bulk for 90 minutes (see Scheme 2) in the presence of dimethylformamide as a polymerisation promoter.^55^ For the copolymerisation between octamethylcyclotetrasiloxane and octaphenylcyclotetrasiloxane, it was necessary to increase the reaction temperature to 90 °C to obtain a homogenous, transparent mixture.

All copolymers were analysed by size exclusion chromatography to assess the apparent molecular mass and the polydispersity. The results are shown in Table 1. In general, the molecular weights are significantly higher than targeted, which suggests inefficient initiation. In addition the polydispersities are relatively large, which indicates that chain rearrangement due to the propagating anion reacting with chains rather than with cyclic monomers is a dominant side-reaction (see Scheme 2). These observations are consistent with literature reports.^55,56^

**Table 1:** Properties of polymers prepared by oxyanionic ring opening polymerisation of D4, T4 and O4

Abbreviation	*M*_n_ (g mol^−1^) target^a^	*M*_n_ b (g mol^−1^)	*M*_w_/*M*_n_^b^	Feed composition	^1^H NMR composition^c^	Non-cyclic fraction^d^
PD	27 000	75 600	1.5	PD	PD	82%
PDT_7525	27 100	58 500	1.5	PD_0.75_PT_0.25_	PD_0.62_PT_0.38_	78%
PDT_5050	26 800	34 500	1.8	PD_0.5_PT_0.5_	PD_0.39_PT_0.61_	84%
PDT_2575	26 200	31 900	2.0	PD_0.25_PT_0.75_	PD_0.19_PT_0.81_	78%
PDO_7525	19 300	34 400	1.8	PD_0.75_PD_0.25_	PD_0.58_PO_0.42_	91%

a Target composition based on the molar ratio between monomers and the initiator.

b Determined by size exclusion chromatography in toluene, using narrow-disperse PDMS standards.

c Based on the ratio between aromatic and aliphatic protons, excluding residual D4, as detailed in the ESI.

d Based on ^1^H NMR spectroscopy.

^1^H NMR spectroscopy of the polydimethylsiloxane homopolymer revealed a significant amount of unreacted D4 (or smaller cyclic compounds) as a signal at 0.75 ppm in addition to the polymer signal at 0.55 ppm (see Fig. S1†). The peak was assigned through comparison with the spectrum of D4 (see Fig. S1†). In addition, the signal disappeared on washing the polymer with methanol, which is known to dissolve low molecular weight cyclic siloxanes.^57^ The relative areas of the two peaks were used for calculating the amount of non-cyclic chains given in Table 1.

**Fig. 1:**
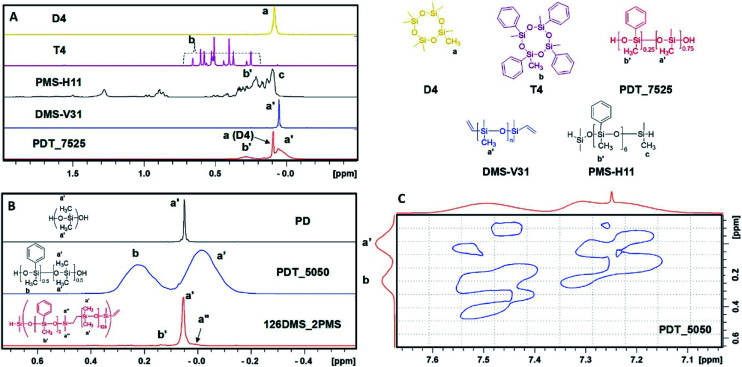
(A) Assigned ^1^H NMR spectra in the range −0.5–1.5 ppm for D4 (yellow), T4 (purple), PMS-H11 (black), DMS-V31 (blue) and PDT_7525 (red). (B) Partial ^1^H NMR spectra of PD, PDT_5050 copolymers and the related multiblock copolymer 126DMS_2PMS prepared by step-growth polymerisation as reported earlier.^51^ (C) Partial NOESY spectrum of PDT_5050 showing through-space correlations between aromatic protons and methyl protons.

The regions of the ^1^H NMR spectra of the copolymers of octamethylcyclotetrasiloxane and tetramethyltetraphenylcyclotetrasiloxane, containing the signals assigned to CH_3_–Si between −0.5 ppm and 2.0 ppm, are shown in Fig. 1A along with spectra from relevant control compounds. Three distinct signals can be discerned: a broad peak between 0.2 ppm and 0.5 ppm, a sharp peak at 0.1 ppm and a broad peak between −0.10 ppm and 0.085 ppm. The sharp peak at 0.1 ppm was assigned to non-reacted D4 or minor cyclics as discussed above. Based on a comparison with a commercial oligo(methylphenylsiloxane), the broad signal between 0.2 ppm and 0.5 ppm was assigned to the methyl protons attached to the phenyl-bearing silicium atom (peaks assigned b′ in Fig. 1A). This assignment was further confirmed by comparing the integrated intensity with that of protons between 7.45 ppm and 7.65 ppm, which can be assigned to two protons in the aromatic ring (not shown). The resulting ratio between aromatic and methyl protons was 2 : 3, as expected. Finally, the broad peak between −0.10 ppm and 0.085 ppm (peaks assigned a′ in Fig. 1A) could be assigned to polydimethylsiloxane segments. This assignment allowed for the calculation of the residual cyclics content and the relative ratio between the dimethylsiloxane and methylphenylsiloxane in the final copolymer. The copolymer spectrum did not show significant amounts of residual tetramethyltetraphenylcyclotetrasiloxane (T4, see Fig. 1A). However, due to substantial overlap between signals from monomer and polymer, a small fraction may remain in the non-purified polymer.

Fig. 1B shows the assigned ^1^H NMR spectra of purified PD and PDT_5050. In addition, the spectrum for a multiblock copolymer of polydimethylsiloxane-polyphenylmethylsiloxane, 126PDMS_2PMS, which was previously prepared using step-growth polymerisation, is shown.^51^ Signals assigned to the dimethylsiloxane segments in the 126PDMS_2PMS multiblock copolymer resemble the signal for the PDMS homopolymer in terms of chemical shift and peak width. However, the corresponding methylsiloxane resonances in the copolymers prepared by ring-opening polymerisation, are significantly broadened. It was hypothesised that the broadening of the signal was due to through-space interactions with aromatic protons on the phenyl ring. The presence of such interactions was confirmed by the acquisition of a NOESY spectrum (see Fig. 1C). Since the Nuclear Overhauser Effect is proportional to the inverse sixth power of the distance,^58^ it follows that all methyl signals are in relatively close vicinity to the phenyl groups for any NOE to be observed. In contrast, the sharp signal observed for the multiblock copolymer (see Fig. 1B) confirms that the majority of the methyl groups are spatially distant from the phenyl groups – as would be expected – due to the very long PDMS blocks and the relatively few phenyl groups. Close inspection of this spectrum reveals a small shoulder at high field (arrow in Fig. 1B, bottom spectrum), which correspond to that of methyl groups that are spatially closer to the phenyl groups (designated a′′ in Fig. 1B, compare to a′ in the spectrum for PDT_5050). The significant intensity of broad methyl peaks and absence of any sharp peaks found in PDT_5050 (see Fig. 1B) and also for PDT_2575 and PDT_7525 (not shown) suggests that the majority of the methyl groups are in close spatial vicinity to one or more phenyl groups. This suggests that the distribution of dimethylsiloxane and methylphenylsiloxane is highly random.

### Elastomer preparation and characterisation

Table 2 shows formulation details of all prepared elastomers. In general, the phenyl-containing copolymers were mixed into the reactive matrix in the amounts given in the table prior to crosslinking. After crosslinking, all the films had a uniform white appearance (not shown), which is a first indication that stable relatively uniform micron-sized domains are formed (not shown). However, increasing the amounts of PDT_2575 and PDO_7525 in the mixture led to macro-phase separation, which indicates poor stabilisation of the domains for these concentrations. PDT_2575 has the highest amount of phenyl groups, while PDO_7525 has a high local density of phenyl groups and so it appears that either too little PDMS or too dissimilar chemistry destabilises the system, as would be expected.

**Table 2:** Details of the elastomer film samples

No.	Copolymer		Sample name	Theoretical phenyl concentration (mmol g^−1^)
	Concentration (phr)	Copolymer		
1	—	—	DMS-V31 (*M*_n_ = 28 000 g mol^−1^) (reference)	0
2	10	PDT_7525	V31 + 10 phr PDT_7525	0.25
3	20		V31 + 20 phr PDT_7525	0.45
4	30		V31 + 30 phr PDT_7525	0.62
5	10	PDT_5050	V31 + 10 phr PDT_5050	0.42
6	20		V31 + 20 phr PDT_5050	0.78
7	30		V31 + 30 phr PDT_5050	1.08
8	5	PDT_2575	V31 + 5 phr PDT_2575	0.55
9	10		V31 + 10 phr PDT_2575	1.01
10	20		V31 + 20 phr PDT_2575	1.40
11	10	PDO_7525	V31 + 10 phr PDO_7525	0.43

Gel fractions of all films were determined from swelling experiments with chloroform, in order to elucidate the amount of bonded (gel fraction) and non-bonded (sol fraction) species in the networks. In general, the gel fraction was found to be in excess of 90% w/w for all films (see Table 3). In addition, the gel fractions of polymer films containing phenyl-containing copolymers were found to be slightly higher than the control. This suggests that there is no inhibitory effect on the crosslinking efficiency due to addition of the copolymers.

**Table 3:** Gel fraction and extract properties

No.	Film designation	Phenyl content^a^/mmol g^−1^	Gel fraction^b^/%	Phenyl copolymer in feed^c^/g g^−1^	Phenyl copolymer in extract^d^/g g^−1^	*M*_p_ 1^e^/g mol^−1^	*M*_p_ 2^f^/g mol^−1^
1	Ref_V31	0	91.5 ± 0.4	0	0	34 600	1040
2	V31 + 10 phr PDT_7525	0.25	94.4 ± 0.5	0.09	0.26 ± 0.07	38 100	1040
3	V31 + 20 phr PDT_7525	0.45	93.3 ± 0.6	0.16	0.26 ± 0.07	47 200	1850
4	V31 + 30 phr PDT_7525	0.62	91.8 ± 0.3	0.23	0.61 ± 0.11	46 700	1040
5	V31 + 10 phr PDT_5050	0.42	94.6 ± 0.3	0.09	0.35 ± 0.08	53 200	1070
6	V31 + 20 phr PDT_5050	0.78	92.8 ± 0.2	0.16	0.48 ± 0.09	48 800	1020
7	V31 + 30 phr PDT_5050	1.08	92.0 ± 0.4	0.23	0.57 ± 0.10	63 200	1040
8	V31 + 5 phr PDT_2575	0.55	94.5 ± 0.3	0.09	0.27 ± 0.07	48 800	1040
9	V31 + 10 phr PDT_2575	1.01	93.7 ± 0.4	0.16	0.48 ± 0.09	51 400	1020
10	V31 + 20 phr PDT_2575	1.40	90.6 ± 0.5	0.23	0.54 ± 0.10	56 900	1040
11	V31 + 10 phr PDO_7525	0.43	95.0 ± 0.7	0.09	0.35 ± 0.09	40 700	1020

a Phenyl content based on the added phenyl copolymer.

b Residual mass fraction of the film after extraction and drying.

c Mass fraction of the added phenyl copolymer.

d Mass fraction of the phenyl copolymer in the extract, calculated from ^1^H NMR.

e Peak molecular weight of the early eluting peak.

f Peak molecular weight relative to the late eluting peak.

Since the phenyl-containing copolymers were physically mixed into the system, it was expected that extraction with a good solvent would lead their removal, while leaving the crosslinked matrix intact. Analysis of extracts from swelling experiments using ^1^H NMR spectroscopy revealed that these were significantly richer in the phenyl-containing copolymer than the feed, and that the amount generally increased with loading (see Table 3). However, the results also show that the amount of phenyl-containing copolymer in the extracts ranges from 0.25 g g^−1^ to 0.60 g g^−1^, which means that a significant amount remains in the matrix. Further analysis using size exclusion chromatography shows that all fractions have a bimodal distribution (see Fig. S2†). The early eluting peak overlaps significantly with the parent phenyl-containing copolymer but has a maximum value at higher retention volume, which indicates a smaller molecular weight. This peak is generally the minor constituent. Similarly, the late eluting peaks overlaps significantly with the crosslinker but peaks at higher retention volume, again indicating lower molecular weight. This is the major constituent. Based on these observations, a tentative explanation for the minor, high molecular weight component is that the longer chains of the phenyl-containing copolymer are highly entangled in the network, why they are harder to extract than the shorter chains. The major, low molecular weight component appears to be related to the crosslinker. One possible explanation is that they stem from the reaction of short hydrosilylated chains with traces of moisture; the crosslinker is a copolymer of dimethylsiloxane and methylhydrosiloxane with around 30% hydrosiloxane groups per chain. Since both the amount of hydrosiloxane groups per chain and the molecular weight follows a distribution, there will be a fraction of short chains with few (or none) hydrosiloxane groups. As such, these are less likely to be incorporated into the network, especially in the presence of trace moisture why they are easily extracted. In addition there may be a small fraction of unreacted monomers and small cycles, since the copolymers were mixed into the reactive mixture without removal of these.

**Fig. 2:**
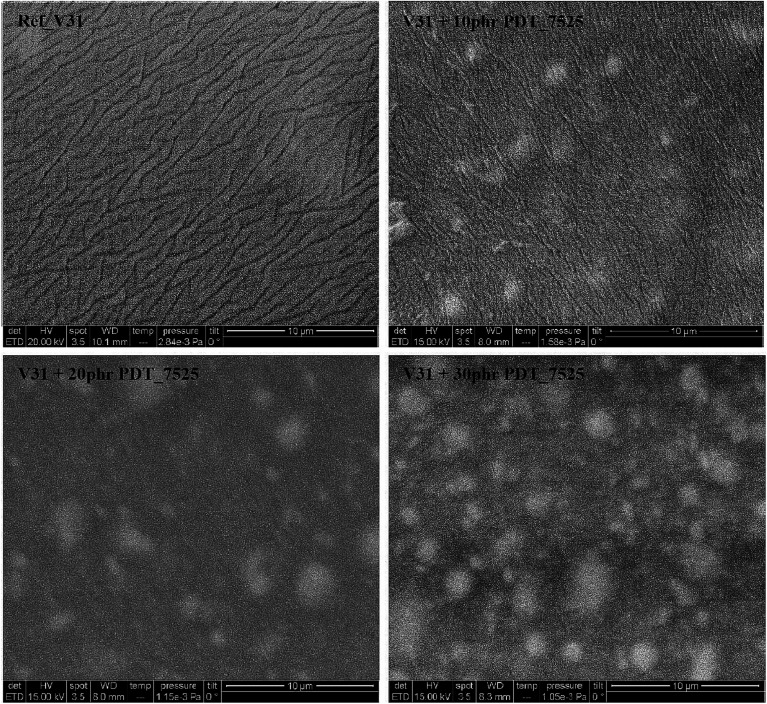
SEM pictures of elastomers with varying loadings of copolymer PDT_7525.

Fig. 2 shows SEM pictures of the crosslinked films containing PDT_7525 in various amounts. In addition is shown the control film without any added copolymer (sample REF_V31). The SEM images of the copolymer elastomers show clearly distinct micron-sized domains (white patches), which appear to be uniformly distributed in the matrix. Increasing the amount of copolymer leads mainly to an increase in the number of domains and to a minor extent to an increase in their size. The intensity difference indicate that these domains are rich in phenyl groups. Such domains are more rigid than the surrounding PDMS matrix and this will reinforce the network,^52^ thus resulting in an elastomer with increased ultimate stress and Young's modulus, as will be discussed in more detail in the subsequent sections. The domain size is significantly smaller than for similar systems where the added phenyl-functionalized silicone polymer results from block copolymers.^52^

### Linear viscoelasticity

**Fig. 3:**
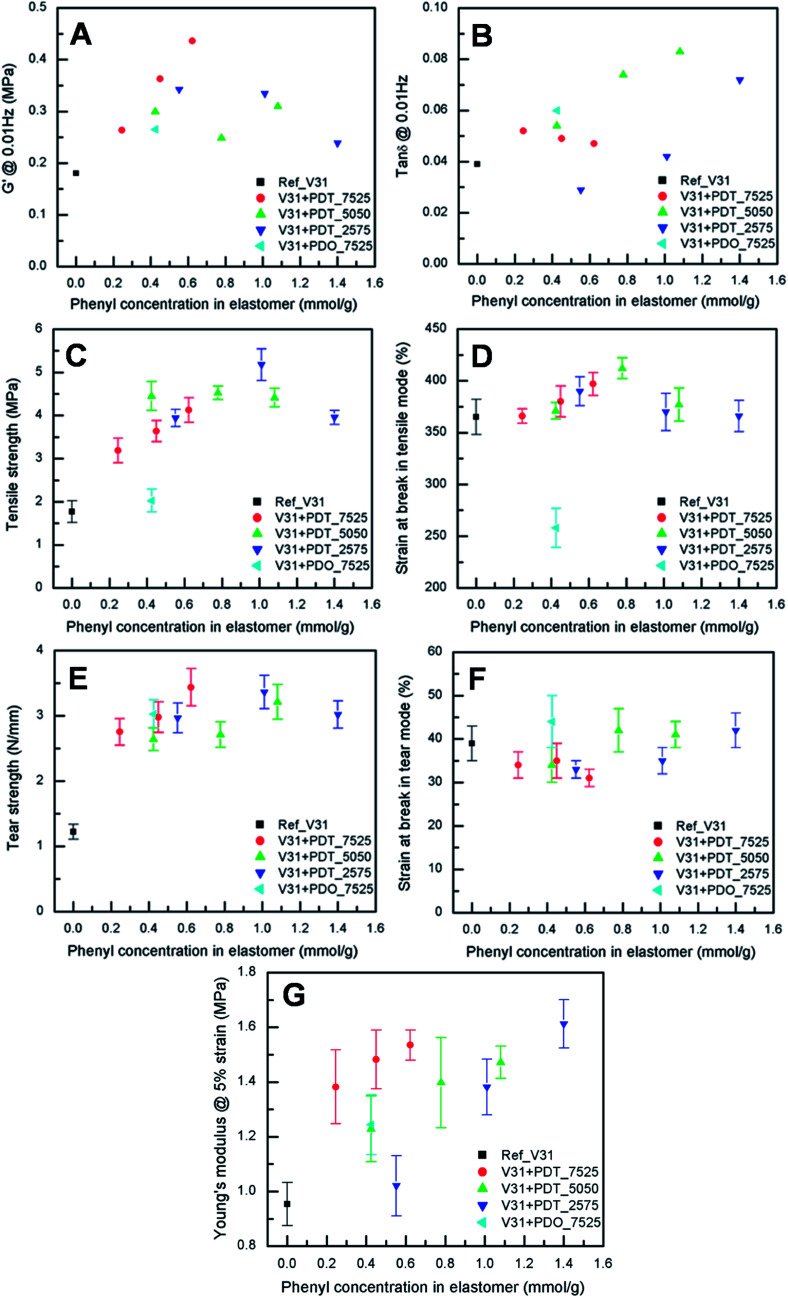
Mechanical properties of elastomers as a function of phenyl content at room temperature. (A) Storage modulus measured at 2% strain and 0.01 Hz. (B) Viscous loss measured at 2% strain and 0.01 Hz. (C) Tensile strength at break. (D) Strain at break under tensile testing conditions. (E) Tear strength. (F) Strain at break under tear testing conditions. (G) Young's modulus at 5% strain.

To evaluate the effect of the increased concentration of the phenyl group on viscoelastic properties, the prepared elastomers were characterised rheologically, as shown in Fig. 3A and B. This is an important investigation to perform for these systems, since aromatic rings may inhibit the platinum catalyst,^59^ which means that the amount of dangling structures – and thus dynamic behaviour – may dominate the networks. However, the elastomers are highly elastic, which indicates a high degree of crosslinking. Thus, the inhibiting nature of the phenyl groups does not affect the final properties of the elastomers significantly. The resulting storage moduli (*G*′) for all copolymer elastomers are higher than for the reference V31 (0.18 MPa) (see Table S1†), which can be attributed to phenyl-rich domains reinforcing the matrix. For all samples, relative viscous losses (as expressed by tan *δ* = *G*′′/*G*′, where *G*′′ is the loss modulus) remain at around or below 9% (see Table S1†), which further supports a high degree of cross-linking and network integrity.

The ageing study of the elastomers shows that the elastomers are very stable over a time of ten months. tan *δ* would immediately reveal if phase separation or other degradation mechanism was taking place since network changes affect tan *δ* immediately. Actually, the only sample that shows any significant change in tan *δ* is the reference silicone elastomer with no phenyl. This elastomer post-cures to some extent and tan *δ* is lowered over time.

### Stress–strain relationship

The results from tensile testing of the cross-linked elastomers are shown in Fig. 3C and D. All the copolymer elastomers show increased ultimate stresses compared to those of reference V31, while the ultimate strains are comparable. Especially, the sample with 10 phr PDT_2575 results in the highest increase in ultimate stress (5.2 ± 0.4 MPa), *i.e.* almost a factor of three increase compared to the reference elastomer (1.8 ± 0.3 MPa) (see Fig. 3C and also Table S2†).

On the other hand, the cross-linked sample with PDO_7525 shows lower ultimate strain, indicating that this copolymer deteriorates network integrity, probably due to the immiscibility of dimethyldiphenylpolysiloxane domains with the matrix. This agrees well with earlier findings indicating that polydiphenylsiloxanes are not compatible with the polydimethylsiloxane matrix.^60^

Interestingly, there seem to be a maximum value of the tensile strength at around 1.0 mmol g^−1^ when the PDT copolymers are used. As indicated by the SEM images (see Fig. 2), increasing the copolymer concentration mainly leads to an increase in the number of reinforcing domains, which remain evenly distributed in the matrix with a near-constant size. At a certain threshold concentration, the domains start to merge, which leads to increase in domain size and ultimately to macro-phase separation which reduces the film integrity.

### Tear strength

Low tear strength is one of the unfavourable but inherent properties of silicone elastomers, and especially for thin films, tearing is a major problem.^17^ Tear strength as a function of phenyl content is shown in Fig. 3E and the strain at tear-induced break is shown in Fig. 3F (see also Table S3†). The presence of phenyl-containing copolymers leads to an increase in tear strength from around 1 N mm^−1^ to around 3 N mm^−1^ at 0.2 mmol g^−1^ (see Fig. 3E). The effect of increasing the overall phenyl content further is relatively limited, although adding PDT_7525 has the largest effect (see Fig. 3E, red data points). Higher phenyl concentration is known to increase tear strength.^61^ In contrast, in high-tear strength silicones, a significant increase in copolymer phase agglomeration size is observed as the elastomer is stretched, indicating that, as the polymer chains of the elastomer matrix become aligned, reorganisation of the copolymer phase occurs, particularly near the point of final rupture.^62^ For this to happen, the surface of the agglomerated copolymer should strongly adhere to the PDMS matrix in the case of the high tear strength samples. This is consistent with the polydimethylsiloxane-polyphenylmethylsiloxane copolymer being able to act as a compatibiliser between phenyl-rich regions and the continuous PDMS phase.

The strain at break on tearing does not change significantly with addition of phenyl-PDMS, presumably because this quantity is mainly dependent on the continuous PDMS matrix.

### Young's modulus

All copolymer containing films have a higher Young's modulus than the reference as seen in Fig. 3G. In general, increasing the phenyl content leads to an increase in the Young's modulus. However, this quantity appears to be dependent on the exact composition of the added copolymer rather than on the absolute phenyl content. For example, using PDT_7525 leads to a modulus of around 1.4 MPa with a phenyl content of 0.2 mmol g^−1^, whereas a phenyl content of around 1.0 mmol g^−1^ is needed to reach the same modulus when using PDT_2575. This allows for fine-tuning of materials properties, as it allows for adjusting the tensile strength (which depends exclusively on the total phenyl content, see Fig. 3C) and the modulus independently.

The effect of increasing the phenyl-concentration using random phenyl-DMS copolymers on Young's modulus is different from that observed when multiblock PDMS-*co*-PPMS elastomers were used.^51^ For the multiblock copolymers the Young's modulus went through a minimum of less than 0.2 MPa at a phenyl concentration of 0.7 mmol g^−1^ and through a maximum of 0.43 MPa around 0.8 mmol g^−1^. Further increasing the phenyl concentration led to a gradual decrease to a value of 0.25 MPa at 2 mmol g^−1^. Thus, the use of random phenyl-DMS copolymers yields a maximum value that can be up to four times as high (1.6 MPa for V31 + 20 phr PDT_2575) and in addition, it is relatively easy to prepare films having a specific value of Young's modulus.

### Dielectric properties and electric breakdown strength

**Fig. 4:**
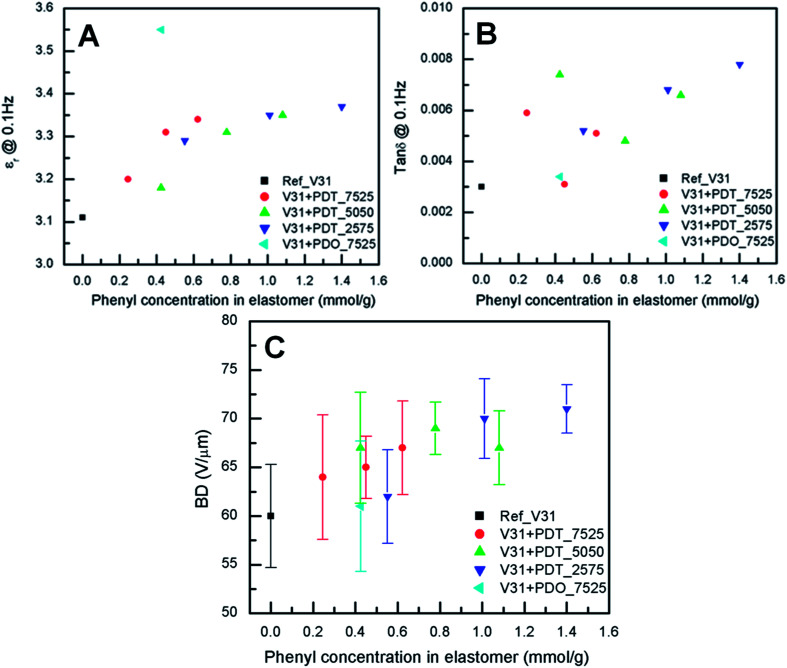
Dielectric properties of elastomers as a function of phenyl concentration for the investigated elastomers at room temperature. (A) Dielectric permittivity at a frequency of 0.1 Hz. (B) Dielectric loss at a frequency of 0.1 Hz. (C) Electrical breakdown strength.

The dielectric properties of the elastomer films are shown in Fig. 4A and B. The relative permittivity of prepared elastomers increases in with increasing phenyl concentration. Fig. 4A shows that the elastomer with 10 phr PDO_7525 has the highest relative permittivity (*ε*_r_ = 3.55) of all samples (see also Table S4†). The relative permittivity of elastomers with PDT copolymers increases up to a concentration of approximately 0.6, where it reaches a value of approximately 3.3. Dielectric losses, as represented by tan *δ*, are relatively low for all cross-linked copolymers, while the reference elastomer (DMS-V31) also shows low tan *δ*.

These values are slightly lower than for the multiblock PDMS-*co*-PPMS elastomers previously reported.^51^ However, the dielectric permittivity of the reference film without phenyl groups was found to be 3.7, (compared to 3.1 found here, see Table S4†) and addition of phenyl groups led to an increase up to 3.9. Thus, the relative increase of the permittivity of the two systems is comparable.

The electrical breakdown strengths of the cross-linked copolymers and the reference elastomer are shown in Fig. 4C. The resulting electrical breakdown strength of the cross-linked copolymers with different phenyl group concentrations all increased compared to the reference elastomer (see Table S4†). The maximum point of electrical breakdown strength (71 ± 2 V μm^−1^) (see Table S4†) was found at the highest measured phenyl concentration of 1.40 mmol g^−1^ with 20 phr PDT_2575. This corresponds to an increase of the electrical breakdown strength of 18% compared to the reference elastomer. The optimum is most likely due to the combination of voltage stabilisation from the charge trapping effect caused by the π-electrons of the phenyl groups^52^ and increased Young's modulus.^63^ The maximum value of the breakdown strength is comparable with that reported earlier using phenyl-containing multiblock copolymers^51^ (72 V μm^−1^), although the relative increase is smaller. Interestingly, for the system based on multiblock copolymers, breakdown strength went through a maximum at a phenyl content of 0.8 mmol g^−1^, whereas no reduction is seen on increasing the phenyl content when random phenyl-PDMS copolymers are used as the source of voltage stabilisers. This difference may be related to the differences in Young's modulus, which also went through a maximum when the multiblock copolymers were used.^51^ Compared to traditional elastomers utilized as dielectric elastomers the absolute value of the electrical breakdown may not be very large but since the copolymers can be mixed with commercially available silicone elastomers the relative increase is also important.

## Summary and conclusions

Silicone-based copolymers with varying concentrations of aromatic groups were synthesised using oxyanionic polymerization by varying the mole ratio between D4 and cyclic phenyl-containing monomers with either one (T4) or two (O4) phenyl groups per silicon. These non-reactive copolymers were blended with reactive PDMS, made into films and cross-linked. The cross-linked films with copolymers containing either T4 or O4 showed an increased storage modulus and lower viscous loss, compared to control films without aromatic groups. Furthermore, the gel fractions were similar, indicating that the network integrity was maintained and the elastomers showed no significant change in electrical or mechanical properties over a storage period of ten months. In addition to having high extensibility, the copolymer elastomers with copolymers containing T4 possessed significantly enhanced tensile strengths of almost 200% of the control, tear strengths of up to 180% of the control as well as Young's moduli of up to 160% compared to the controls. Adding a copolymer containing O4 also led to an increase in tear strength and Young's modulus, whereas a decrease in the ultimate strain indicated a deteriorated network. Furthermore, relative permittivity increased with increasing phenyl concentration, without compromising dielectric loss. In particular, mixing a copolymer containing O4 proved effective in increasing the relative permittivity. All of the elastomers with copolymers containing T4 had higher electrical breakdown strength than the pure reference PDMS elastomer. The maximum electrical breakdown strength was found for a phenyl concentration of 1.40 mmol g^−1^, which was 18% higher than the reference elastomer. In conclusion, it was found that polysiloxanes containing a random distribution of methyl and phenyl substituents may form uniform dispersions in a crosslinked PDMS matrix. This affects the mechanical and electrical properties of the films in a predictable matter. In general, addition of copolymers of dimethylsiloxane with no more than 50% mol mol^−1^ methylphenylsiloxane leads to an increase in moduli, strength, dielectric permittivity and breakdown voltage. The addition of a copolymer with more than 50% mol mol^−1^ methylphenylsiloxane or with diphenylsiloxane instead of methylphenylsiloxane leads to an increase in some properties but may lead to a decrease in others at higher concentrations. This can be explained by the existence of a dispersion of phenyl-rich domains in a continuous PDMS matrix. Stable, separate micron-sized domains lead to increased mechanical and electrical strengths. However, destabilisation caused by increasing concentration or polymer incompatibility may lead to macro-phase separation, which can lead to defects in the material.

## Conflicts of interest

There are no conflicts to declare.

## Supplementary Material

RA-008-C8RA02314J-s001
