# Supplementary material for: Enhancing the electro-mechanical properties of polydimethylsiloxane elastomers through blending with poly(dimethylsiloxane-co-methylphenylsiloxane) copolymers
Source: RSC Adv. 2018 Jun 25;8(41):23077–88. doi: 10.1039/c8ra02314j (PMC9081550; doi:10.1039/c8ra02314j)
Supplement: RA-008-C8RA02314J-s001 [file RA-008-C8RA02314J-s001.pdf]

Electronic Supplementary Material (ESI) for RSC Advances.
This journal is © The Royal Society of Chemistry 2018

Electronic Supplementary information

Enhancing the electro-mechanical properties of polydimethylsiloxane elastomers through blending with poly(dimethylsiloxane-co-methylphenylsiloxane) copolymers

Peter Jeppe Madsen, Liyun Yu, Sarah Boucher, Anne Ladegaard Skov*

Danish Polymer Centre, Department of Chemical and Biochemical Engineering, Technical University of Denmark, Søltofts Plads Building 227, 2800 Kgs. Lyngby, Denmark

*Corresponding author: Tel.: +45 45252825. Fax: +45 45882258. E-mail: al@kt.dtu.dk

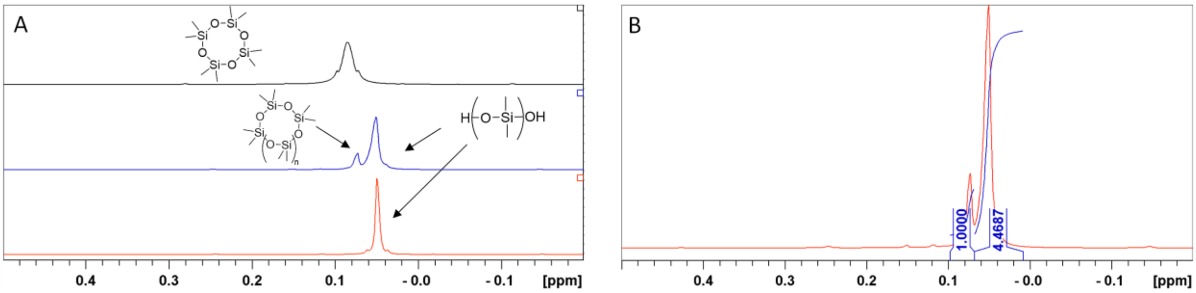

Figure S1. A) Black spectrum, top: ^{1}H NMR of D4. Blue spectrum, middle: ^{1}H NMR of PDMS prepared by the oxyanionic polymerisation of D4. Signals assigned to cyclic and linear components are marked. Red Spectrum, bottom: ^{1}H NMR of PDMS prepared by the oxyanionic polymerisation of D4 after removing cyclic components by washing with methanol. **B**) Integrals used in the evaluation of the ratio between cyclic and linear chains.

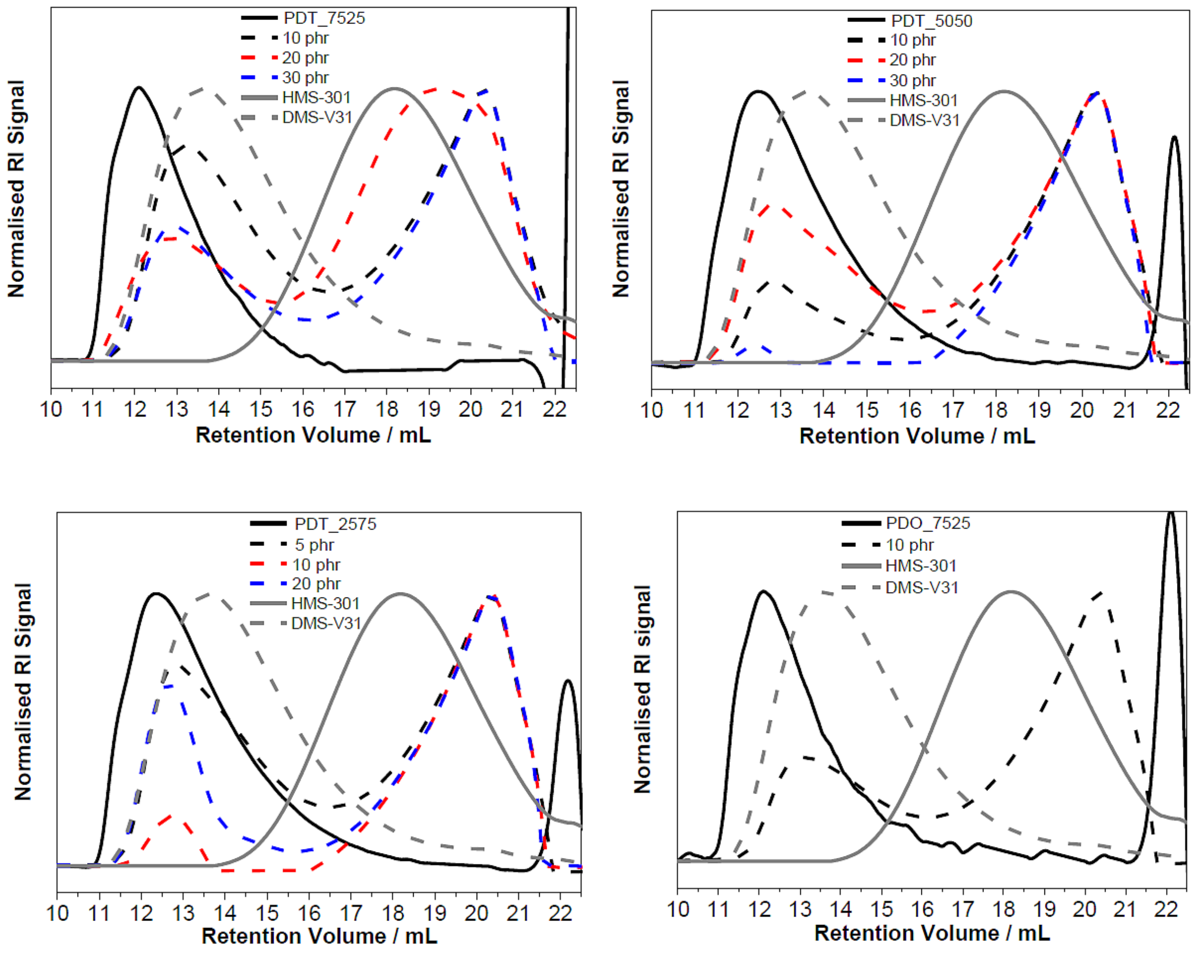

Figure S2. Size exclusion chromatograms of film extracts and starting materials.

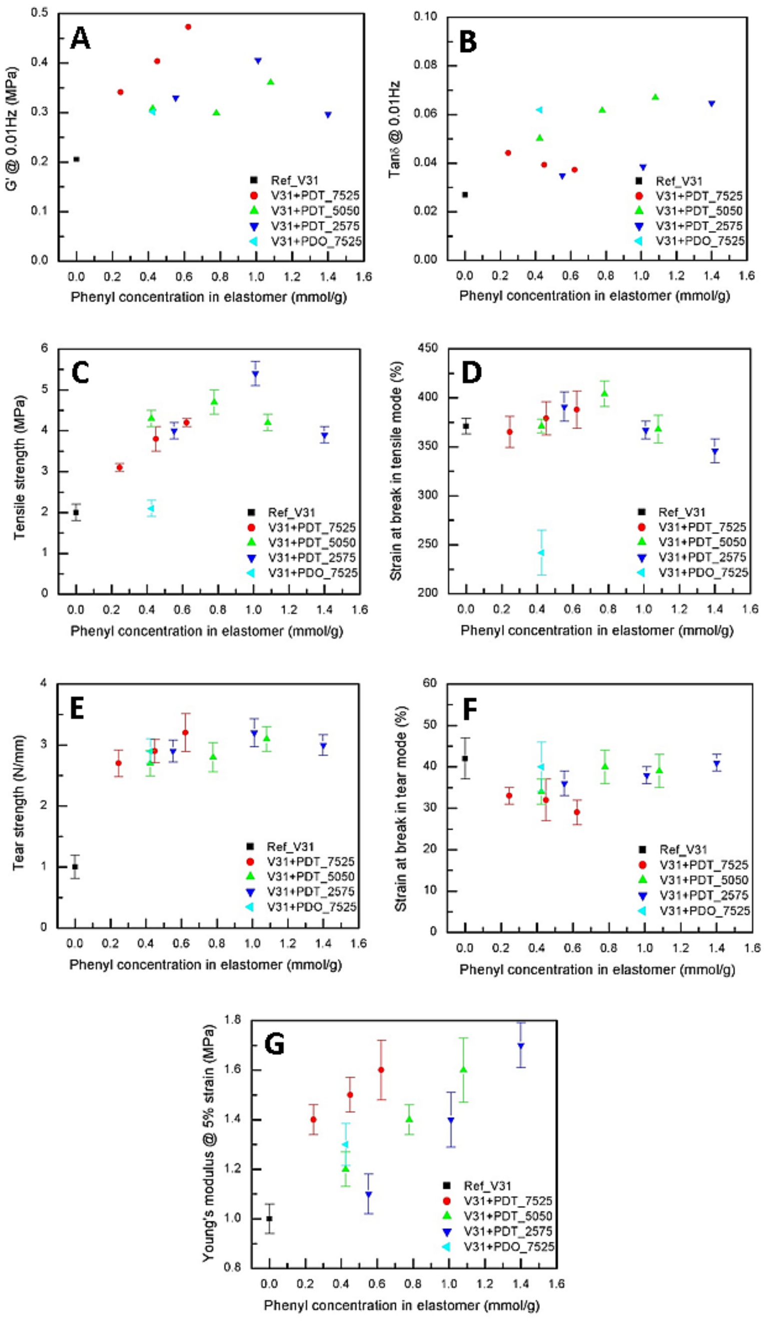

Figure S3. Mechanical properties of elastomers (10 months) as a function of phenyl content at room temperature. **A**) Storage modulus measured at 2% strain and 0.01 Hz. **B**) Viscous loss measured at 2% strain and 0.01 Hz. **C**) Tensile strength at break. **D**) Strain at break under tensile testing conditions. **E**) Tear strength. **F**) Strain at break under tear testing conditions. **G**) Young's modulus at 5 % strain.

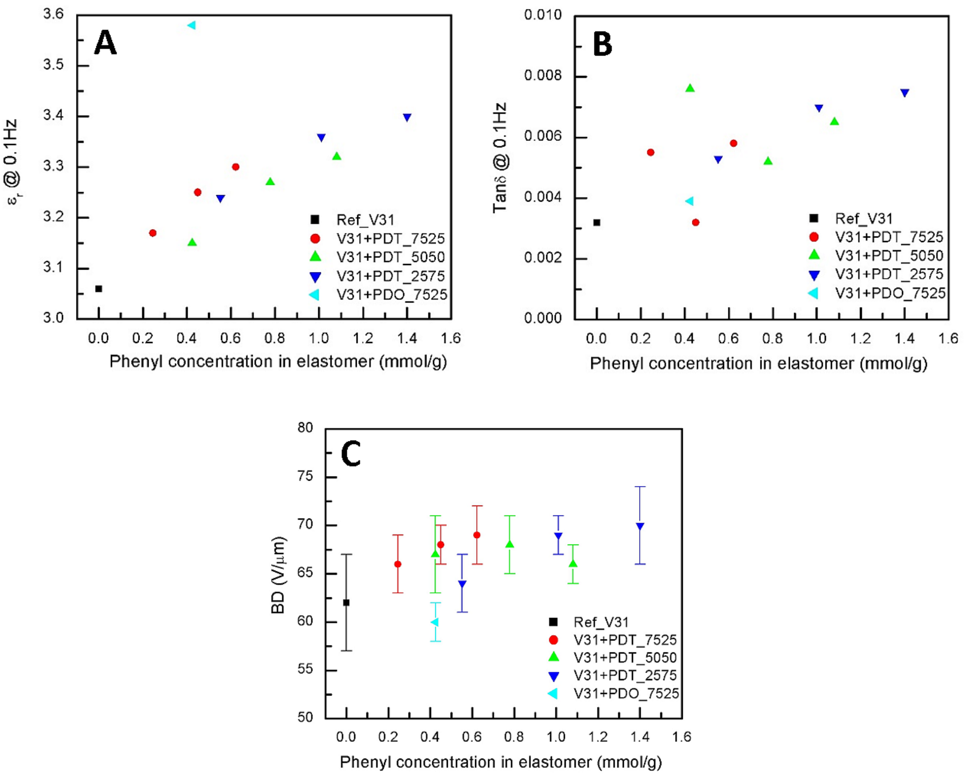

Figure S4. Dielectric properties of elastomers (10 months) as a function of phenyl concentration for the investigated elastomers at room temperature. **A**) Dielectric permittivity at a frequency of 0.1 Hz. **B**) Dielectric loss at a frequency of 0.1 Hz. **C**) Electrical breakdown strength.

Calculation of copolymer composition from ^{1}H NMR

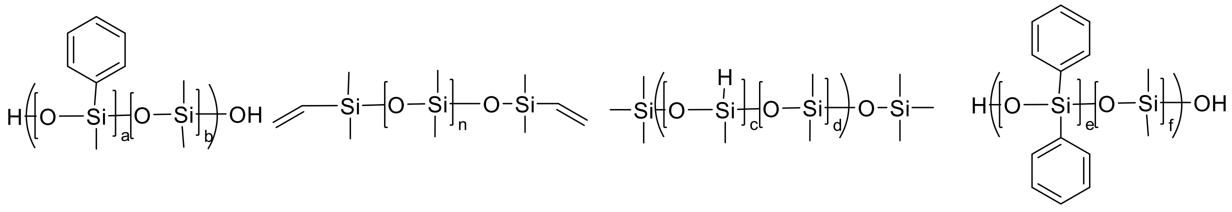

PDT_ba DMS-V31 HMS-301 PDO_fe

Scheme S1. Chemical structure of PDT_ba, DMS-V31, HMS-301 and PDO_fe.

NMR signals between 6.8 and 7.8 ppm are assigned to the phenyl group of T (five protons). Signals between 0.1 and 0.5 ppm can be assigned to the methyl groups of T, and signals

between -0.1 and 0.09 ppm can be assigned to methyl groups of polymerised D (six protons from D). The sharp signal at 0.1 ppm can be assigned to the monomer or small homocycles. From the relative intensities of signals assigned to phenyl, I_{Ph} and signals assigned to methyl groups of polymerised D, the ratio between a and b can be calculated:

Equation 1

$$\frac{a}{b} = \frac{\frac{I_{Ph}}{h_{Ph}}}{\frac{I_{Me}^{D}}{h_{Ph}^{D}}}$$

where h denotes the amount of protons that constitute the signal.

Calculation of the non-cyclic fraction from ^{1}H NMR

The non-cyclic fraction can be calculated from the signals assigned to cyclic components at 0.1 ppm and the non-cyclic fractions at higher and lower fields:

Equation 2

$$\text{Non} - \text{cyclic fraction} = 1 - \frac{\frac{I_{Me}^{Dc}}{h_{Me}^{Dc}}}{\frac{I_{Me}^{Dc}}{h_{Me}^{Dc}} + \frac{I_{Me}^{D}}{h_{Me}^{D}} + \frac{I_{Me}^{T}}{h_{Me}^{T}}}$$

where Dc denotes cyclic dimethylsiloxane, and the notation otherwise is as before.

Calculation of molar and mass ratios for PT_aD_b copolymer extracts

The intensity of the methyl groups assigned to the phenyl-containing copolymer can be expressed from the intensity of the aromatic signal, a and b (or f and g), and the amount of protons that give rise to the signals.

The ratio between the intensity of protons assigned to methyl groups and the intensity of protons assigned to phenyl groups is related to the molar ratio of the constituents as:

Equation 3

$$\frac{I_{Me}^{Tot}}{I_{Ph}} = \frac{h_1' n_{PTaDb}^{Ph} + h_2 n_{PTaDb}^{NP} + h_3 n_{DMS-V31} + h_4 n_{HMS-301}}{h_1 n_{PTaDb}^{Ph}}$$

$$= \frac{h_1' a n_{PTaDb} + h_2 b n_{PTaDb} + h_3 n_{DMS-V31} + h_4 n_{HMS-301}}{h_1 a n_{PTaDb}} \Rightarrow$$

$$\frac{I_{Me}^{Tot}}{I_{Ph}} - \frac{h_1'}{h_1} - \frac{h_2 b}{h_1 a} = \frac{h_3 n_{DMS-V31}}{h_1 a n_{PTaDb}} + \frac{h_4 n_{HMS-301}}{h_1 a n_{PTaDb}}$$

Without detailed knowledge of the relative amounts of DMS-V31 and HMS-301 in the extracts, the molar ratio cannot be calculated. However, on the assumption that the concentration of either DMS-V31 or HMS-301 is zero, an expression for the ratio can be obtained:

Equation 4

$$\left(\frac{I_{Me}^{Tot}}{I_{Ph}} - \frac{h_1'}{h_1} - \frac{h_2 b}{h_1 a}\right)\frac{h_1 a}{h_{3/4}} = \frac{n_Y}{n_{PTaDb}}$$

where Y is DMS-V31 or HMS-301 and $h_{3/4}$ is h_3 for no HMS-301 or h_4 for no DMS-V31.

For h_4, an average value based on the composition should be used:

Equation 5

$$h_4 n_{HMS-301} = h_4 c n_{HMS-301} + h_4' d n_{HMS-301} = (h_4 c + h_4' d) n_{HMS-301}$$

$$= (h_4 c + h_4'(1-c)) n_{HMS-301}$$

which equates to 3*0.3+6*0.7=5.1 using the manufacturer's composition.

The assumption that all DMS-V31 is incorporated into the network is favoured by the fact that HMS-31 is used in excess. This is backed to some extent by the SEC data.

The mass ratio can be obtained from the molecular mass of the repeating unit:

Equation 6

$$\frac{n_Y}{n_{PTaDb}} = \frac{m_Y/M_Y}{m_{PTaDb}/M_{PTaDb}} \Rightarrow \frac{m_Y}{m_{PTaDb}} = \frac{n_Y M_Y}{n_{PTaDb} M_{PTaDb}}$$

Calculation of molar and mass ratios for PTTeDf copolymer extracts

The corresponding ratios for the extracts that contain the PTTeDf copolymer can be evaluated in a similar manner, in that the ratio between the intensity of protons assigned to methyl groups and the intensity of protons assigned to phenyl groups is related to the molar ratio of the constituents as:

Equation 7

$$\frac{I_{Me}^{Tot}}{I_{Ph}} = \frac{h_6 n_{PTTeDf}^{NP} + h_3 n_{DMS-V31} + h_4 n_{HMS-301}}{h_5 n_{PTTeDf}^{Ph}}$$

$$= \frac{h_6 f n_{PTTeDf} + h_3 n_{DMS-V31} + h_4 n_{HMS-301}}{h_5 e n_{PTTeDf}} \Rightarrow$$

$$\frac{I_{Me}^{Tot}}{I_{Ph}} - \frac{h_6 f}{h_5 e} = \frac{h_3 n_{DMS-V31}}{h_5 e n_{PTTeDf}} + \frac{h_4 n_{HMS-301}}{h_5 e n_{PTTeDf}}$$

If there is no DMS-V31 or no HMS-301, this reduces to a similar expression as before:

Equation 8

$$\left(\frac{I_{Me}^{Tot}}{I_{Ph}} - \frac{h_6 f}{h_5 e}\right)\frac{h_5 e}{h_{3/4}} = \frac{n_Y}{n_{PTTeDf}}$$

The mass ratios can be evaluated as before, using the mass of the repeating group of PTT_eD_f:

Equation 9

$$\frac{n_Y}{n_{PTTeDf}} = \frac{m_Y/M_Y}{m_{PTTeDf}/M_{PTTeDf}} \Rightarrow \frac{m_Y}{m_{PTTeDf}} = \frac{n_Y M_Y}{n_{PTTeDf} M_{PTTeDf}}$$

Estimation of uncertainty on mass ratios

The uncertainty on the mass ratios were estimated by assuming uncertainty on the NMR integrals of ± 10 % and a value of 6 for h_3 (no HMS-301) and 5.1 for h_4 (no HMS-301). The range can thus be defined by calculating a maximum and a minimum value for the molar ratio, using Equation 6 and Equation 7. The uncertainty is defined as ± half of this range.

Table S1. Linear viscoelastic properties of the investigated elastomers at 2% strain and 0.01 Hz at room temperature.

Sample	G’ @0.01Hz (MPa)	tanδ @0.01Hz
DMS-V31 (reference)	0.18	3.9e-02
V31 + 10 phr PDT_7525	0.26	5.2e-02
V31 + 20 phr PDT_7525	0.36	4.9e-02
V31 + 30 phr PDT_7525	0.44	4.7e-02
V31 + 10 phr PDT_5050	0.30	5.4e-02
V31 + 20 phr PDT_5050	0.25	7.4e-02
V31 + 30 phr PDT_5050	0.31	8.3e-02
V31 + 5 phr PDT_2575	0.34	2.9e-02
V31 + 10 phr PDT_2575	0.34	4.2e-02
V31 + 20 phr PDT_2575	0.24	7.2e-02
V31 + 10 phr PDO_7525	0.27	6.0e-02

Table S2. Young’s modulus, tensile strength and strain at break of the investigated elastomers at room temperature.

Sample	Y@5%strain (MPa)	Tensile strength (MPa)	Strain at break in tensile mode (%)
DMS-V31 (reference)	1.0±0.1	1.8±0.3	365±17
V31 + 10 phr PDT_7525	1.4±0.1	3.2±0.3	366±7
V31 + 20 phr PDT_7525	1.5±0.1	3.6±0.2	380±15
V31 + 30 phr PDT_7525	1.5±0.1	4.1±0.3	397±11
V31 + 10 phr PDT_5050	1.2±0.1	4.4±0.3	371±8

V31 + 20 phr PDT_5050	1.4±0.2	4.5±0.2	412±10
V31 + 30 phr PDT_5050	1.5±0.1	4.4±0.2	377±16
V31 + 5 phr PDT_2575	1.0±0.1	3.9±0.2	390±14
V31 + 10 phr PDT_2575	1.4±0.1	5.2±0.4	370±18
V31 + 20 phr PDT_2575	1.6±0.1	4.0±0.2	366±15
V31 + 10 phr PDO_7525	1.2±0.1	2.0±0.3	258±19

Table S3. Tear strength of the investigated elastomers at room temperature.

Sample	Tear strength (N/mm)	Strain at break in tear mode (%)
DMS-V31 (reference)	1.2±0.1	39±4
V31 + 10 phr PDT_7525	2.8±0.2	34±3
V31 + 20 phr PDT_7525	3.0±0.2	35±4
V31 + 30 phr PDT_7525	3.4±0.3	31±2
V31 + 10 phr PDT_5050	2.6±0.2	34±4
V31 + 20 phr PDT_5050	2.7±0.2	42±5
V31 + 30 phr PDT_5050	3.2±0.3	41±3
V31 + 5 phr PDT_2575	3.0±0.2	33±2
V31 + 10 phr PDT_2575	3.4±0.3	35±3
V31 + 20 phr PDT_2575	3.0±0.2	42±4
V31 + 10 phr PDO_7525	3.0±0.2	44±6

Table S4. Dielectric properties and electrical breakdown strength of the investigated elastomers at room temperature.

Sample	ε_r @0.1Hz	tanδ @0.1Hz	BD (V/μm)
DMS-V31 (reference)	3.11	3.0e-03	60±5
V31 + 10 phr PDT_7525	3.20	5.9e-03	64±6
V31 + 20 phr PDT_7525	3.31	3.1e-03	65±3
V31 + 30 phr PDT_7525	3.34	5.1e-03	67±5
V31 + 10 phr PDT_5050	3.18	7.4e-03	67±6
V31 + 20 phr PDT_5050	3.31	4.8e-03	69±3
V31 + 30 phr PDT_5050	3.35	6.6e-03	67±4
V31 + 5 phr PDT_2575	3.29	5.2e-03	62±5
V31 + 10 phr PDT_2575	3.35	6.8e-03	70±4
V31 + 20 phr PDT_2575	3.37	7.8e-03	71±2
V31 + 10 phr PDO_7525	3.55	3.4e-03	61±7

Table S5. Linear viscoelastic properties of the aged elastomers (10 months) at 2% strain and 0.01 Hz at room temperature.

Sample	G’ @0.01Hz (MPa)	tanδ @0.01Hz
DMS-V31 (reference)	0.20	2.7e-02
V31 + 10 phr PDT_7525	0.34	4.4e-02
V31 + 20 phr PDT_7525	0.40	3.9e-02
V31 + 30 phr PDT_7525	0.47	3.7e-02
V31 + 10 phr PDT_5050	0.31	5.0e-02

V31 + 20 phr PDT_5050	0.30	6.2e-02
V31 + 30 phr PDT_5050	0.36	6.7e-02
V31 + 5 phr PDT_2575	0.33	3.5e-02
V31 + 10 phr PDT_2575	0.40	3.9e-02
V31 + 20 phr PDT_2575	0.30	6.5e-02
V31 + 10 phr PDO_7525	0.30	6.2e-02

Table S6. Young's modulus, tensile strength and strain at break of the aged elastomers (10 months) at room temperature.

Sample	Y@5%strain (MPa)	Tensile strength (MPa)	Strain at break in tensile mode (%)
DMS-V31 (reference)	1.0±0.1	2.0±0.2	371±8
V31 + 10 phr PDT_7525	1.4±0.1	3.1±0.1	365±16
V31 + 20 phr PDT_7525	1.5±0.1	3.8±0.3	379±17
V31 + 30 phr PDT_7525	1.6±0.1	4.2±0.1	388±19
V31 + 10 phr PDT_5050	1.2±0.1	4.3±0.2	371±7
V31 + 20 phr PDT_5050	1.4±0.1	4.7±0.3	404±13
V31 + 30 phr PDT_5050	1.6±0.1	4.2±0.2	368±14
V31 + 5 phr PDT_2575	1.1±0.1	4.0±0.2	391±15
V31 + 10 phr PDT_2575	1.4±0.1	5.4±0.3	367±9
V31 + 20 phr PDT_2575	1.7±0.1	3.9±0.2	346±12
V31 + 10 phr PDO_7525	1.3±0.1	2.1±0.2	242±23

Table S7. Tear strength of the aged elastomers (10 months) at room temperature.

Sample	Tear strength (N/mm)	Strain at break in tear mode (%)
DMS-V31 (reference)	1.0±0.2	42±5
V31 + 10 phr PDT_7525	2.7±0.2	33±2
V31 + 20 phr PDT_7525	2.9±0.2	32±5
V31 + 30 phr PDT_7525	3.2±0.3	29±3
V31 + 10 phr PDT_5050	2.7±0.2	34±3
V31 + 20 phr PDT_5050	2.8±0.2	40±4
V31 + 30 phr PDT_5050	3.1±0.2	39±4
V31 + 5 phr PDT_2575	2.9±0.2	36±3
V31 + 10 phr PDT_2575	3.2±0.2	38±2
V31 + 20 phr PDT_2575	3.0±0.2	41±2
V31 + 10 phr PDO_7525	2.9±0.2	40±6

Table S8. Dielectric properties and electrical breakdown strength of the aged elastomers (10 months) at room temperature.

Sample	ε_r @0.1Hz	tanδ @0.1Hz	BD (V/μm)
DMS-V31 (reference)	3.06	3.2e-03	62±5
V31 + 10 phr PDT_7525	3.17	5.5e-03	66±3
V31 + 20 phr PDT_7525	3.25	3.2e-03	68±2
V31 + 30 phr PDT_7525	3.30	5.8e-03	69±3
V31 + 10 phr PDT_5050	3.15	7.6e-03	67±4
V31 + 20 phr PDT_5050	3.27	5.2e-03	68±3

V31 + 30 phr PDT_5050	3.32	6.5e-03	66±2
V31 + 5 phr PDT_2575	3.24	5.3e-03	64±3
V31 + 10 phr PDT_2575	3.36	7.0e-03	69±2
V31 + 20 phr PDT_2575	3.40	7.5e-03	70±4
V31 + 10 phr PDO_7525	3.58	3.9e-03	60±2
